# Importance of the Collagen Adhesin Ace in Pathogenesis and Protection against *Enterococcus faecalis* Experimental Endocarditis

**DOI:** 10.1371/journal.ppat.1000716

**Published:** 2010-01-08

**Authors:** Kavindra V. Singh, Sreedhar R. Nallapareddy, Jouko Sillanpää, Barbara E. Murray

**Affiliations:** 1 Division of Infectious Diseases, Department of Internal Medicine, University of Texas Medical School, Houston, Texas, United States of America; 2 Center for the Study of Emerging and Re-emerging Pathogens; University of Texas Medical School, Houston, Texas, United States of America; 3 Department of Microbiology and Molecular Genetics, University of Texas Medical School, Houston, Texas, United States of America; Dartmouth Medical School, United States of America

## Abstract

Ace is an adhesin to collagen from *Enterococcus faecalis* expressed conditionally after growth in serum or in the presence of collagen. Here, we generated an *ace* deletion mutant and showed that it was significantly attenuated versus wild-type OG1RF in a mixed infection rat endocarditis model (*P*<0.0001), while no differences were observed in a peritonitis model. Complemented OG1RFΔ*ace* (pAT392::*ace*) enhanced early (4 h) heart valve colonization versus OG1RFΔ*ace* (pAT392) (*P* = 0.0418), suggesting that Ace expression is important for early attachment. By flow cytometry using specific anti-recombinant Ace (rAce) immunoglobulins (Igs), we showed *in vivo* expression of Ace by OG1RF cells obtained directly from infected vegetations, consistent with our previous finding of anti-Ace antibodies in *E. faecalis* endocarditis patient sera. Finally, rats actively immunized against rAce were less susceptible to infection by OG1RF than non-immunized (*P* = 0.0004) or sham-immunized (*P* = 0.0475) by CFU counts. Similarly, animals given specific anti-rAce Igs were less likely to develop *E. faecalis* endocarditis (*P* = 0.0001) and showed fewer CFU in vegetations (*P* = 0.0146). In conclusion, we have shown for the first time that Ace is involved in pathogenesis of, and is useful for protection against, *E. faecalis* experimental endocarditis.

## Author Summary

*Enterococcus faecalis* was recognized as a common cause of infective endocarditis (IE) by the early 1900s. It is still third in community-onset IE, but is the second most common cause of hospital-associated IE. Complications due to *E. faecalis* IE include congestive heart failure, septic emboli and death and current management involves a combination of antimicrobials, often with surgery. Emergence of antimicrobial resistance has created the need for alternative strategies (such as immunoprophylaxis) that target *in vivo* expressed virulence-associated surface proteins. One such *E. faecalis* protein is Ace, which is antigenic during human IE and mediates attachment of *E. faecalis* cells to host extracellular matrix proteins collagen and laminin. Using a rat model, we now show that *ace* contributes to *E. faecalis* IE pathogenesis and demonstrate that Ace is expressed at high levels during IE even though produced at low levels under laboratory conditions; both active and passive immunization based on the collagen-binding domain of Ace conferred significant protection against IE. These observations, along with data that human antibodies against Ace inhibit collagen adherence of *E. faecalis*, indicate that Ace is an important virulence-associated factor and a promising target for prophylactic and possibly therapeutic strategies against *E. faecalis* IE.

## Introduction

Enterococci are gram-positive cocci of intestinal origin first reported as a cause of infective endocarditis (IE) in 1899 [1]. They were recognized as the 3^rd^ most common cause of IE as early as the 1920's, and have remained the 3^rd^ most common cause of community onset IE since then with *Enterococcus faecalis* accounting for >90% of isolates from enterococcal IE when identified to the species level [1],[2],[3],[4],[5]. Over the past 20 years, enterococci have also become the 2^nd^–3^rd^ most common organisms isolated from nosocomial (healthcare-associated) infections including UTIs, bacteremia, intraabdominal and wound infections, endocarditis, sepsis in neonates, among others [1],[3]. Indeed, among causes of endocarditis, enterococci (predominantly *E. faecalis*) have been variably reported as the #1 and #2 cause [6],[7]. Since healthcare-associated infections, particularly those caused by antibiotic resistant bacteria, result in enormous increases in hospital stays and costs, enterococci clearly represent an important drain on healthcare dollars. In one study, the attributable mortality of enterococcal bacteremia was 31% [8], emphasizing the clinical, not just the financial, seriousness of these infections.

The first step in infective endocarditis is vascular tissue colonization, which can be mediated by cell-wall anchored adhesins such as MSCRAMMs (for microbial surface components recognizing adhesive matrix molecules) [9] of gram-positive bacteria. Our previous *in silico* analyses of the *E. faecalis* genome identified a family of genes encoding MSCRAMM-like proteins containing one or more regions of *ca.* 150 aa segments with deviant Ig-like fold(s), characteristic of the *Staphylococcus aureus* MSCRAMMs [10]. One of these, called Ace (for Adhesin to collagen of *E. faecalis*), has been studied in detail. Genetic and biochemical analyses showed that Ace mediates adherence of *E. faecalis* cells to bovine and rat collagen type I (CI), human collagen type IV (CIV), and mouse laminin [11],[12],[13], as well as human dentin [14].

Crystal structure analysis of the ligand-binding segment of Ace showed that the Ace A domain is composed of two sub-domains, N1 and N2, each adopting an Ig-like fold [15]. Subsequent point and truncation mutation analyses suggested that Ace binds to collagen by a mechanism called the “Collagen Hug”[15], a variant of the “Dock, Lock and Latch” ligand-binding mechanism shown for *Staphylococcus epidermidis* fibrinogen (Fg) adhesin SdrG [16],[17]. The *ace* gene is ubiquitous [18] in *E. faecalis* and conserved among diverse isolates albeit with at least four variants due to variation in the number of repeats of the B domain [19]. Conditional *in vitro* production of Ace (i.e., markedly enhanced production after growth at 46°C, growth in brain-heart infusion plus 40% serum (BHIS) or growth in the presence of collagen versus growth in BHI broth at 37°C) by different strains correlates with conditional adherence of these *E. faecalis* strains to collagens and laminin [19],[20]. Most sera from patients with *E. faecalis* IE show reactivity with rAce, indicating that different strains express Ace during human infection and that it is antigenic *in vivo* [19]. Furthermore, anti-Ace antibodies (affinity purified from human serum or animals immunized with rAce) were shown to inhibit *in vitro* adherence of *E. faecalis* strains to collagen and laminin [11],[19]. In a recent study, anti-Ace40 (ligand-binding A-domain of Ace) monoclonal antibodies were shown to completely inhibit binding of Ace40 to human CI and collagen type VI and inhibited binding of Ace-coated fluorescent beads to epithelial cell lines, thus suggesting Ace as a potential therapeutic target antigen against *E. faecalis* infections [21].

In the present study, we have studied the role of Ace in the pathogenesis of *E. faecalis* endocarditis by generating an *ace* deletion mutant in *E. faecalis* strain OG1RF, by complementing this mutant (OG1RFΔ*ace*), by comparing these isogenic strains with OG1RF for adherence to various extracellular matrix (ECM) proteins and for their ability to infect aortic valves in a rat endocarditis model. Finally, we also determined the importance of Ace as a protective antigen against experimental endocarditis in a rat model by using active and passive immunization.

## Results

### Characterization of the Δ*ace* mutant and complementation construct

**Figure 1:**
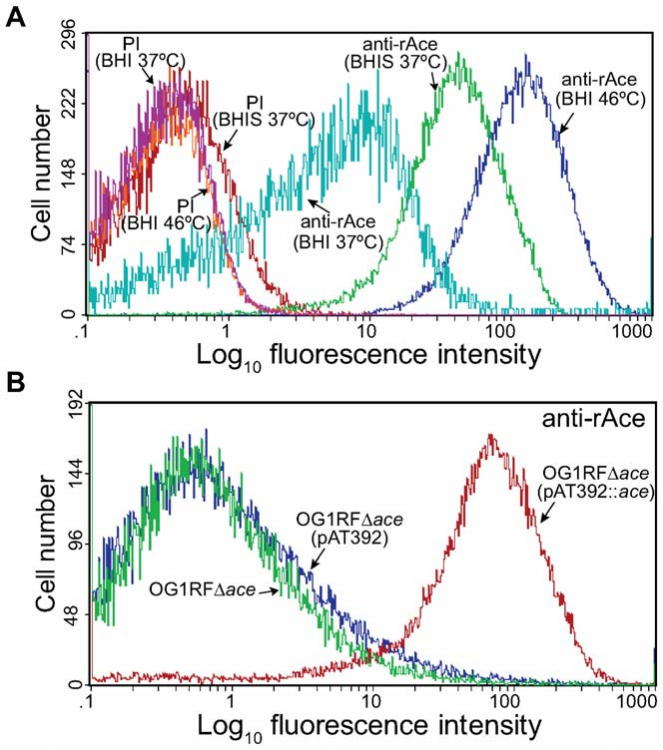
Flow cytometry analysis of cell surface expression of Ace by *E. faecalis* OG1RF, its isogenic *ace* deletion mutant and its *in trans* complemented *ace* deletion mutant. (A) Comparison of the effect of growth for 10 h under different conditions on expression levels of Ace in OG1RF using both pre-immune Igs (PI) and anti-rAce Igs. (B) Analysis of Ace expression by the OG1RF *ace* deletion mutant and the effect of its *in trans* complementation. OG1RFΔ*ace*, *ace* deletion mutant; OG1RFΔ*ace* (pAT392::*ace*), complemented *ace* deletion mutant; OG1RFΔ*ace* (pAT392), *ace* deletion mutant with the empty vector. Reactivity to affinity-purified specific anti-rAce Igs is shown for each isogenic strain. Bacteria were analyzed using side scatter as the threshold for detection. Binding by specific anti-rAce Igs is indicated as log fluorescence intensity on the X-axis. For each histogram, 50,000 events of bacterium-sized particles were counted.

Our previous disruption mutant of *ace* was found to be unstable *in vivo* (see below). We therefore constructed an allelic replacement *ace* deletion mutant of OG1RF (TX5467, OG1RFΔ*ace*::*cat*; resistant to chloramphenicol 10 µg/ml). Deletion of *ace* from OG1RF was verified by sequencing confirming the correct deletion of *ace* from −23 to + 2200 (including the RBS, complete *ace* gene, and 34 bp downstream of *ace*), and by pulsed field gel electrophoresis (PFGE) and hybridizations (Table 1). Growth (OD_600_) of the Δ*ace* mutant was similar to wild-type (WT) OG1RF in BHI (data not shown). We have previously shown, using western blotting and RT-PCR, that *ace* is expressed at higher levels when grown in BHIS at 37°C or in BHI at 46°C [11] than in BHI at 37°C. Here, we assessed surface localization of Ace in OG1RF at 10 h using flow cytometry analyses with affinity-purified anti-rAce Igs. The mean fluorescence intensity levels for different culture conditions increased progressively with cells grown in BHI at 37°C, BHIS at 37°C and BHI at 46°C (Figure 1A), consistent with our previous western and immunofluorescence microscopy data [11],[20]. The percentages (%) of Ace-expressing cells in BHIS cultures of OG1RF, OG1RFΔ*ace*, OG1RFΔ*ace* (pAT392) (empty vector control), and OG1RFΔ*ace* (pAT392::*ace*) (complementation) were >70%, <5%, <5%, and >90%, respectively, demonstrating the inability of OG1RFΔ*ace* to produce Ace and the efficient complementation of OG1RFΔ*ace* by pAT392::*ace*. In these experiments, pAT392-containing strains were grown without added gentamicin, the same conditions that we used for preparing inocula for the rat endocarditis experiments. When BHIS was supplemented with gentamicin, expression of Ace increased to >95% of cells in OG1RFΔ*ace* (pAT392::*ace*) (Figure 1B), likely due to improved plasmid stability (see below).

**Table 1:** Bacterial strains and plasmids used in this study.

Strains/Plasmids	Relevant characteristics^a^	Reference or source
Strains		
*E. faecalis*		
OG1RF	Laboratory strain; Rif ^r^, Fus^r^, Chl^s^, Gen^s^, Kan^s^	[61]
TX5256	*ace* insertion disruption mutant of OG1RF; Rif ^r^, Fus^r^, Kan^r^, Chl^s^, Gen^s^	[11]
TX5467	OG1RFΔ*ace*::*cat*, *ace* deletion mutant of OG1RF; Rif ^r^, Fus^r^, Kan^s^, Chl^r^, Gen^s^	This study
TX5647	TX5467 harboring pTEX5646 (for complementation with the *ace* gene); Rif ^r^, Fus^r^, Chl^r^, Gen^r^	This study
TX5648	TX5467 harboring pAT392 (control for complementation); Rif ^r^, Fus^r^, Chl^r^, Gen^r^	This study
*E. coli*		
DH5α	*E. coli* host strain for routine cloning	Stratagene
XL1-Blue	*E. coli* host strain for routine cloning	Stratagene
LMG194	*E. coli* strain for expression of recombinant proteins	Invitrogen
TX5254	LMG194 (pBAD::*ace*); 1008 bp OG1RF *ace* (coding for complete A domain) cloned into pBAD/HisA expression vector; Amp^r^	[11]
TX5428	DH5α (pTEX5428); Chl^r^, Gen^r^	This study
TX5646	XL1-Blue (pTEX5646); Gen^r^	This study
Plasmids		
pAT392	Shuttle expression vector (Gen^r^ Spc^r^ *oriR*_pUC_ *oriR*_pAMβ1_ *oriT*_RK2_ *P_2_*)	[51]
pTEX5500ts	Shuttle plasmid, ts in Gram^+^ hosts; Chl^r^, Gen^r^	[50]
pTEX5646	Construct for complementation; a 2,186-bp fragment containing *ace* cloned into pAT392 downstream of the P2 promoter	This study
pTEX5428	Plasmid for *ace* deletion with flanking regions of the *ace* gene cloned on either side of the *cat* gene into pTEX5500ts; Chl^r^, Gen^r^	This study

*^a^*Chl, chloramphenicol; Fus, fusidic acid; Gen, gentamicin; Kan, kanamycin; Rif, rifampicin; and ts, temperature-sensitive. Superscript “s” designates sensitivity and “r” designates resistance; “r” is defined for enterococci as MIC >500 for Gen and >2000 for Kan.

**Figure 2:**
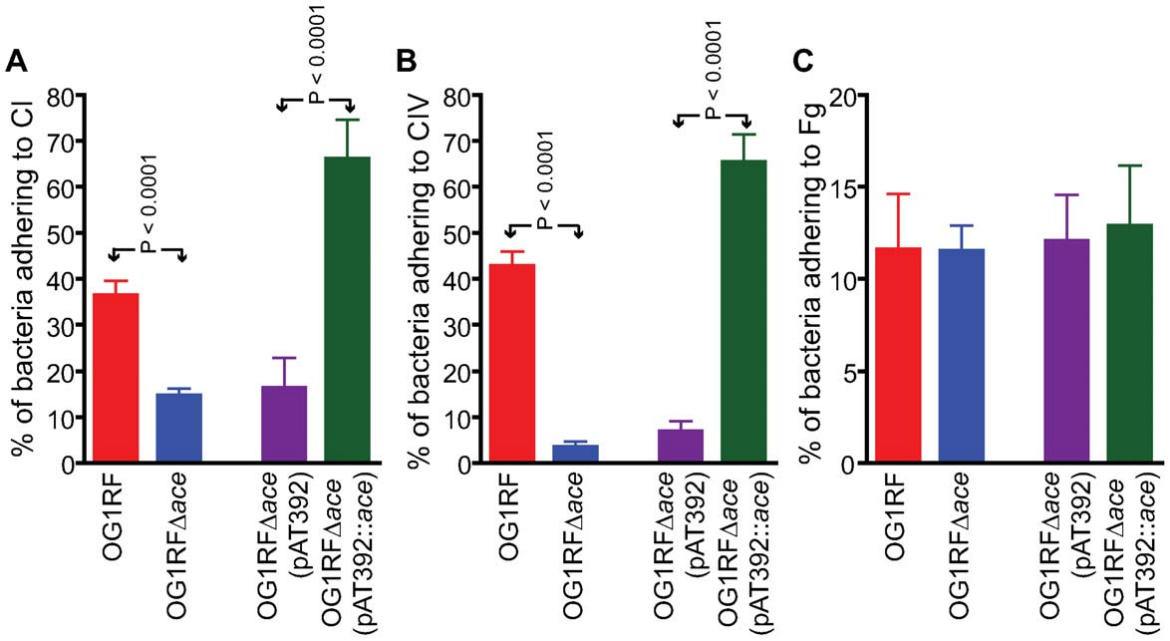
Adherence of *E. faecalis* OG1RF and its derivatives to immobilized collagens. (A) Adherence to collagen type I (CI). (B) Adherence to collagen type IV (CIV). (C) Adherence to fibrinogen (Fg). Mean % of cells adhering ± SD from two independent experiments representing 12 wells/sample are shown.

OG1RF and its isogenic Δ*ace* mutant as well as the complementation constructs were tested for their ability to adhere to immobilized ECM proteins and BSA. Consistent with our previous demonstration of adherence of OG1RF to CI, CIV and Fg after growth in BHIS at 37°C and to CI and CIV after growth in BHI at 46°C (but not in BHI [11] at 37°C), we observed here that OG1RF adhered to CI and CIV when grown in BHIS at 37°C, unlike OG1RFΔ*ace* which showed markedly reduced adherence to CI (from ∼36 to 15%) (Figure 2A), and CIV (43 to 3%) (Figure 2B), but no change in adherence to Fg ((Figure 2C). This corroborates our earlier data with a mutant with an insertional disruption of *ace* [11],[20]. Introduction of the *ace* gene *in trans* into OG1RFΔ*ace* resulted in even greater adherence to collagens than WT (>1.5-fold higher), whereas OG1RFΔ*ace* electroporated with pAT392 retained its reduced adherence phenotype (Figure 2); these results are consistent with Ace expression data from flow cytometry analysis.

### *In vivo* surface expression of Ace

**Figure 3:**
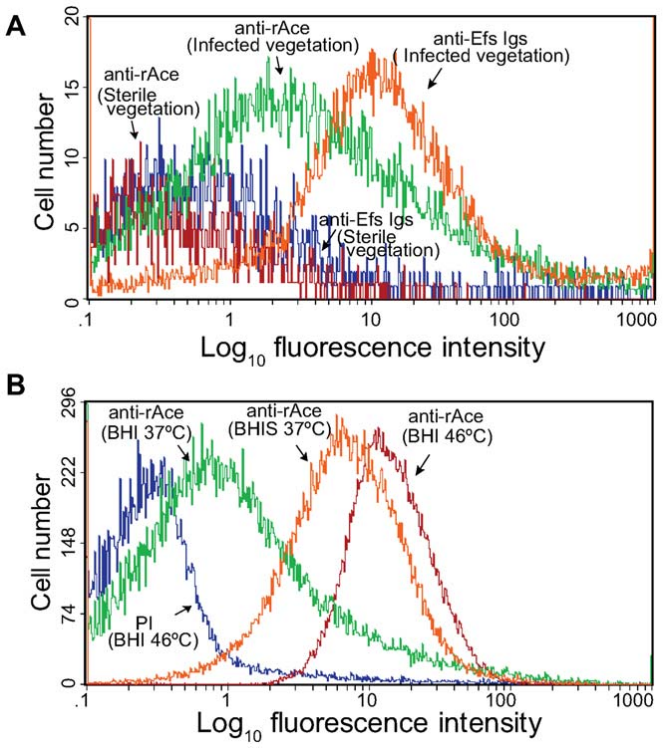
Flow cytometry analysis of Ace surface expression by *E. faecalis* OG1RF cells derived from vegetations of rat experimental infective endocarditis. (A) Surface expression of Ace by bacterium sized particles derived from vegetations. Vegetations were produced as described in methods, and some rats were injected (*i.v.*) with BHI-grown OG1RF. Processed sterile and infected vegetations were incubated with specific anti-Ace A-domain Igs or Igs purified from antiserum raised against heat-killed *E. faecalis* strain HH22-whole-cells (anti-Efs), followed by incubation with R-phycoerythrin-conjugated antibody. Specific binding by anti-Ace or anti-Efs antibodies is indicated as log fluorescence intensity on the X*-*axis. Each histogram represents 5,000 (non-infected vegetations) to 25,000 (infected vegetations) events of bacterium-sized particles. (B) For comparison, OG1RF cells grown *in vitro* in BHI at 37°C (inoculum used for infection) and 46°C (the growth condition that exhibited most *in vitro* Ace expression) as well as in BHIS at 37°C, stained with pre-immune Igs and anti-Ace and the same batch of R-phycoerythrin-conjugated secondary antibody, are shown in panel B.

To determine if Ace is produced during infection, we performed flow cytometry analyses on extracts directly processed from IE vegetations infected with OG1RF grown in BHI at 37°C. Forward and side scatter pattern analyses of particles from processed vegetations and comparisons with those from *in vitro* grown OG1RF cells indicated that most of the gated particles detected by flow cytometry are likely OG1RF bacterial cells, thus confirming the removal of the majority of host tissue particles from the vegetations during the processing steps described in methods. Sterile processed vegetations from non-infected rats probed with anti-rAce-specific Igs (negative control) showed labeling of a minor fraction (<3%) of bacterium-sized particles (Figure 3A), while processed vegetations from OG1RF infected rats probed with Igs from an antiserum raised against formalin-killed *E. faecalis* strain HH22-whole-cells (positive control) bound 85% of bacterium-sized particles, further indicating that the majority of these particles were *E. faecalis* cells (Figure 3A). Affinity-purified anti-rAce-specific Igs bound to ∼40 to 45% of bacterium-sized particles from different rat endocarditis vegetations infected with OG1RF (Figure 3), demonstrating that Ace is actively expressed in host vegetations during IE.

### *In vivo* testing of WT OG1RF and *ace* mutants in a mixed infection competition assay

Although our initial mixed-infection competition experiments showed a clear advantage for the WT over an *ace* disruption mutant TX5256 [11],[20] to develop IE in rat model (data not shown), subsequent experiments identified instability of this single cross-over *ace* disruption mutant during *in vivo* growth. Hence, we generated an OG1RFΔ*ace* mutant for further *in vivo* testing.

In an initial mono-infection experiment (n = 2) with our *ace* deletion mutant (OG1RFΔ*ace*), TX5467, we first determined the expression of *cat* (encoding chloramphenicol acetyl transferase) in OG1RFΔ*ace*, which carries this chloramphenicol resistance marker gene in place of *ace,* by analyzing individual colonies for chloramphenicol resistance and by high stringency hybridization using intragenic *cat* and *ace* DNA probes. We found that ∼10% of the colonies recovered from vegetations were chloramphenicol (10 µg/ml) susceptible even though they were *cat* probe positive and *ace* probe negative, indicating that, although the *cat* gene was present, silencing of its expression had occurred in these colonies. Previously, it has been shown that some antibiotic resistance genes of *Escherichia coli* are silenced *in vivo*; specifically, expression of an intact antibiotic resistance gene was switched off during the course of gut colonization in pigs, a phenomenon suggested to be helpful for bacterial fitness [22]. Therefore, for our mixed infection animal experiments, all the results reported here are based on high stringency hybridization with *ace* and *cat* probes using ∼200 CFUs/rat vegetation for all the rats used. Of interest, we also tested for chloramphenicol resistance but did not observe further *cat* silencing.

**Figure 4:**
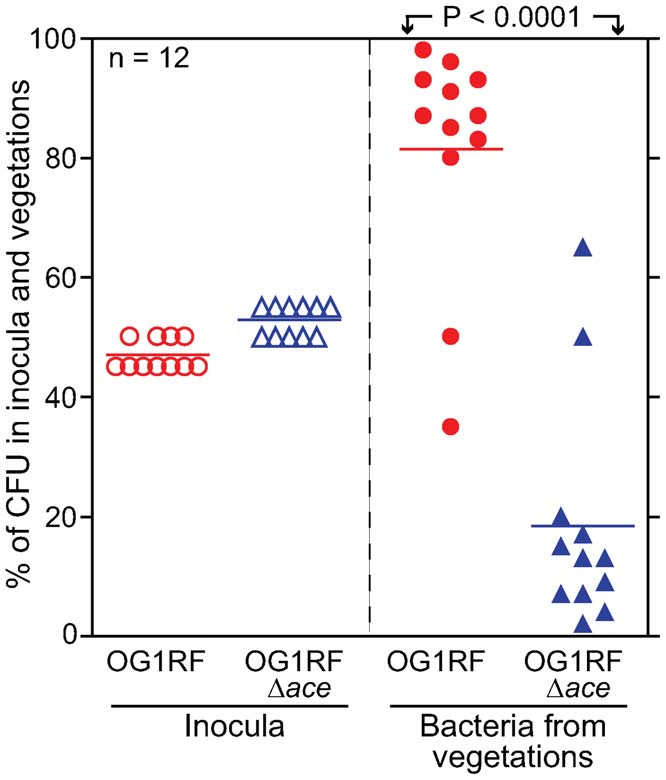
*E. faecalis* OG1RF and OG1RFΔ*ace* (TX5467) in a competition (mixed infection) assay in the rat endocarditis model. Percentages of OG1RF and OG1RFΔ*ace* present in inocula and recovered from vegetations 72 h post infection of 12 rats are shown. Horizontal bars represent the means (*P*<0.0001 by paired *t* test) for percentages of total bacteria in the vegetations of OG1RF versus OG1RFΔ*ace*. Empty circles and empty triangles represent percentages of OG1RF and OG1RFΔ*ace* in inocula, respectively, while solid circles and solid triangles represent percentages of OG1RF and OG1RFΔ*ace* in vegetations, respectively.

In the mixed infection competition assay, all 12 rats were infected with an approximately 1∶1 mixture (as predicted by OD_600_) of BHI-grown OG1RF (determined geometric mean (GM) CFU 3.8×10^7^/rat, representing 47% of the inoculum): OG1RFΔ*ace* (GM CFU 4.4×10^7^/rat, representing 53% of the inoculum) (Figure 4). Bacterial CFUs from vegetations on aortic valves were recovered at ∼72 h from all 12 rats and are shown in Figure 4. The mean percentage (%) of OG1RF in the total CFU of bacteria recovered was 81.5% versus 18.5% for OG1RFΔ*ace* (*P*<0.0001), thus demonstrating a clear advantage of OG1RF versus OG1RFΔ*ace* at 72 h for heart valve colonization in rats. The mean virulence index or competitive index [23],[24], which is a sensitive measure of the relative degree of virulence attenuation of a particular mutant in a mixed infection with the WT strain, was calculated using the equation shown in Materials and Methods. The mean virulence index of the *ace* mutant relative to WT in vegetations was 0.077; this indicates that *ace* has an important role in this endovascular infection.

### *In vivo* testing of the complemented OG1RFΔ*ace* (pAT392::*ace*) construct and OG1RFΔ*ace* (pAT392) in the mono-infection model

**Figure 5:**
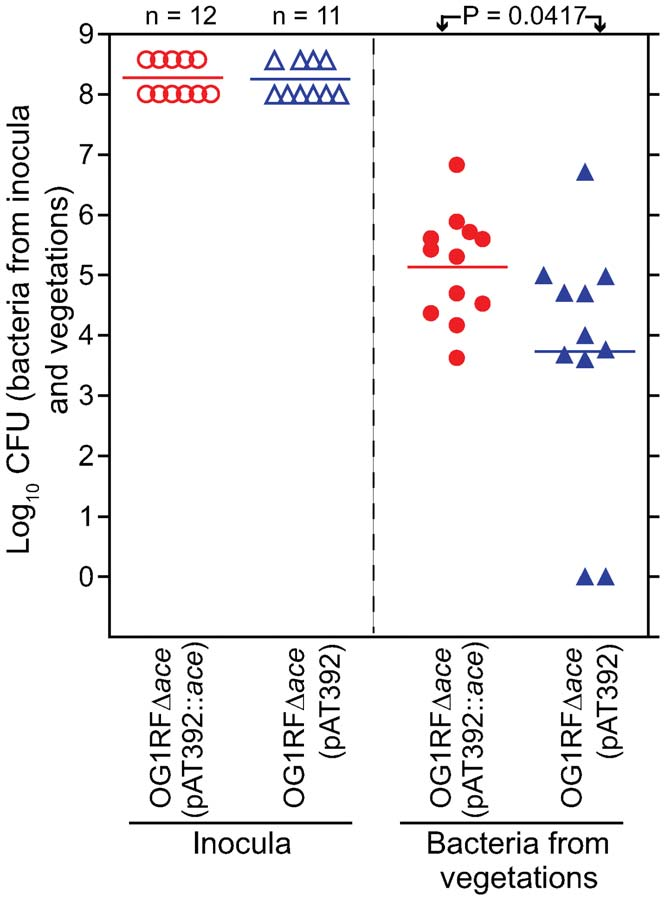
Complemented OG1RFΔ*ace* (pAT392::*ace*) [TX5647] and OG1RFΔ*ace* (pAT392) [TX5648] in early (mono-infection) colonization of aortic valves in the rat model. In the panel on the left, empty circles and empty triangles represent OG1RFΔ*ace* (pAT392::*ace*) and OG1RFΔ*ace* (pAT392) inocula, respectively. In the panel on the right, solid circles and solid triangles represent OG1RFΔ*ace* (pAT392::*ace*) and OG1RFΔ*ace* (pAT392) in vegetations, respectively. Data are expressed as log_10_ CFU/gm recovered from the vegetations 4 h post infection of 12 rats (OG1RFΔ*ace* (pAT392::*ace*) and 11 rats (OG1RFΔ*ace* (pAT392), respectively. Horizontal bars represent the geometric mean titers. Significantly enhanced (by a mean ± SD increase of 1.4±0.6 log_10_ CFU/gm) vegetation titer by OG1RFΔ*ace* (pAT392::*ace*) versus OG1RFΔ*ace* (pAT392) (*P* = 0.0417) by unpaired *t* test for mean log_10_CFU/gm is shown.

In initial mono-infection experiments with complementation constructs and testing 24 h after inoculation, we observed loss of the plasmid from cells recovered from vegetations, with 94%-98% loss from OG1RFΔ*ace* (pAT392::*ace*) (7 rats) and 14%–100% loss from OG1RFΔ*ace* (pAT392) (8 rats) (data not shown). We also tried growing both constructs in BHIS supplemented with gentamicin for the preparation of inocula for infection, but *in vivo* loss of the plasmid still occurred 24 h after inoculation. To minimize *in vivo* growth time and to determine the role of Ace in the early stage of valve colonization in rats, we tested both OG1RFΔ*ace* (pAT392::*ace*) and OG1RFΔ*ace* (pAT392) in the rat model 4 h after inoculation. Two independent mono-infection experiments were done and the combined results are shown in Figure 5. Rats inoculated with OG1RFΔ*ace* (pAT392::*ace*) (n = 12) showed 1.4±0.6 log_10_ more CFU/gm than OG1RFΔ*ace* (pAT392) (n = 11) (*P* = 0.0417) (Figure 5), thus demonstrating that Ace has a significant role in early colonization of heart valves in *E. faecalis* rat IE. Reduced time *in vivo* also resulted in much less loss of the plasmid from each construct. In the case of OG1RFΔ*ace* (pAT392::*ace*), gentamicin susceptible colonies were recovered in only 2/12 rats (2/2 colonies from one and 3/3 colonies from the other were gentamicin susceptible), while with OG1RFΔ*ace* (pAT392), 3–100% of colonies (among 8–48 tested) recovered from 5/11 rats were gentamicin susceptible. These results corroborated the above described complementation of *ace* surface expression (Figure 1B) and restoration of *in vitro* adherence of OG1RFΔ*ace* (pAT392::*ace*) to CI and CIV to similar levels as observed for OG1RF.

### Active immunization with rAce and *in vivo* protection against *E. faecalis* experimental IE in rats

**Figure 6:**
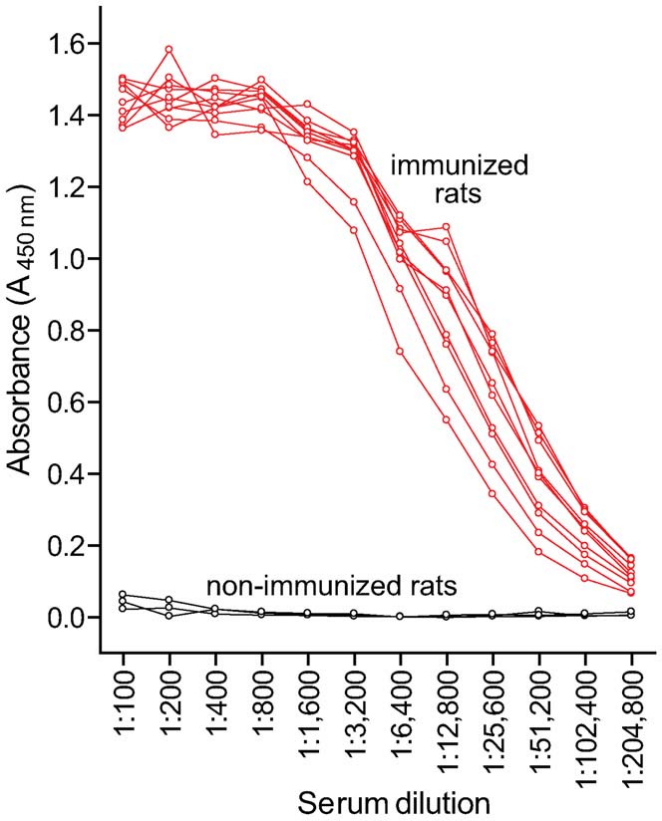
Comparison of serum anti-Ace titers in immunized and non-immunized rats. Rats were immunized and boosted twice with 100 μg rAce. Antibody levels were measured by ELISA. Mean serum anti-Ace titers were plotted for each antibody dilution tested.

**Figure 7:**
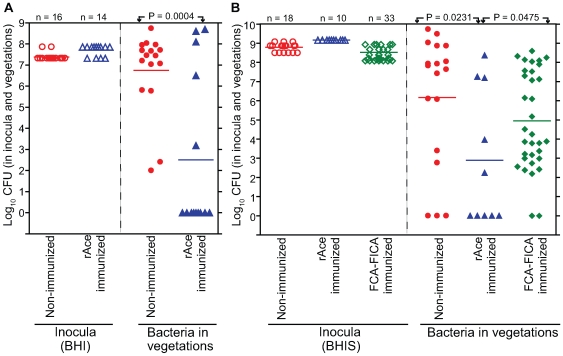
rAce active-immunization in rat endocarditis model. (A) BHI-grown OG1RF. In the panel on the left, empty circles and empty triangles represent OG1RF used for non-immunized and rAce active-immunized rats, respectively. In the panel on the right, solid circles and solid triangles represent OG1RF recovered from the vegetations, 48 h post infection. Horizontal bars represent the geometric means. Significantly fewer rats were infected by OG1RF in rAce active-immunized (5/16) versus non-immunized (16/16) (*P* = 0.0001 by Fisher's exact test). Vegetations showed 4.2±1.0 log_10_ more OG1RF CFU/gm (mean ± SD) from non-immunized versus rAce active-immunized rats (*P* = 0.0004, by unpaired *t* test). (B) BHIS-grown OG1RF. In the panel on the left, empty circles, empty triangles and empty diamonds represent OG1RF used for non-immunized, rAce active-immunized and sham-immunized rats, respectively. In the panel on the right, solid circles, solid triangles and solid diamonds represent OG1RF recovered from the vegetations, 48 h post infection. Horizontal bars represent the geometric means. Significantly reduced vegetation bacterial counts of OG1RF in rAce active-immunized (n = 18), versus non-immunized (n = 10) (*P* = 0.0231) and versus sham-immunized (n = 33) (*P* = 0.0475) by unpaired *t* test are shown. Rats (non-immunized) showed a mean ± SD increase of 3.2±1.3 log_10_ OG1RF CFU/gm from vegetations versus rAce active-immunized rats while rats (FCA-FICA immunized) showed a mean ± SD increase of 2.0±1.0 log_10_ OG1RF CFU/gm from vegetations versus rAce active-immunized rats.

Since Ace was found to be an important virulence factor in rat experimental IE, an Ace-specific immune response might hinder the development of IE. To study this, rats were vaccinated *s.c.* thrice with 99% pure 100 µg rAce or PBS or Freund's complete adjuvant – Freund's incomplete adjuvant (FCA – FICA) and were challenged with 10^7^ to 10^9^ CFU of *E. faecalis* OG1RF per rat (see methods). Comparison of anti-Ace antibody levels of 10 immunized and three non-immunized animals by ELISA showed that all 10 immunized rats tested had high levels of anti-Ace titers (1: >50,000), whereas no anti-Ace antibodies were detected in any of the three control rats (Figure 6). Sixteen of 16 no-treatment control rats (100%) developed *E. faecalis* endocarditis after challenge with 10 times the ID_50_ of BHI-grown OG1RF compared with 5 of 14 rats (35%) in the rAce active-immunization group (*P* = 0.0001) (Figure 7A). The no-treatment control rats showed a mean of 4.2±1.0 log_10_ more CFU/gm than the rAce active-immunized (*P* = 0.0004) in vegetations recovered from heart valves. In an independent experiment, in order to mimic *in vivo* growth conditions more closely and because we had found that Ace is expressed at higher levels by OG1RF when grown in BHIS [11], we used BHIS-grown OG1RF for the preparation of inocula. rAce (n = 10) rats were inoculated with a higher inoculum of 1.4×10^9^ CFU/rat (∼100 times the ID_50_), while non-immunized controls (n = 18) were inoculated with 3.1×10^8^ –1.1×10^9^ CFU/rat. Fifteen of 18 non-immunized control animal (83%) developed *E. faecalis* endocarditis compared with 5 of 10 rats (50%) in the rAce-immunized group with a mean ± standard deviation (SD) increase of 3.2±1.3 log_10_ CFU (*P* = 0.0231) in the non-immunized group (Figure 7B), showing significantly reduced bacterial titers in rAce-immunized group even when given a higher inoculum. Thirty-three sham (FCA - FICA)-immunized control group rats inoculated with 1.2–3.8×10^8^ CFU/rat also showed significantly higher bacterial counts with a mean ± SD increase of 2.0±1.0 log_10_ CFU/gm (*P* = 0.0475) versus the rAce active-immunized treatment group (n = 10) (Figure 7B). These results demonstrate reproducibility of *in vivo* protection by active immunization with rAce against *E. faecalis* experimental endocarditis in rats using two different growth conditions to prepare the inocula.

### *In vivo* protection against *E. faecalis* experimental endocarditis in rats using anti-rAce Igs for passive immunization

**Figure 8:**
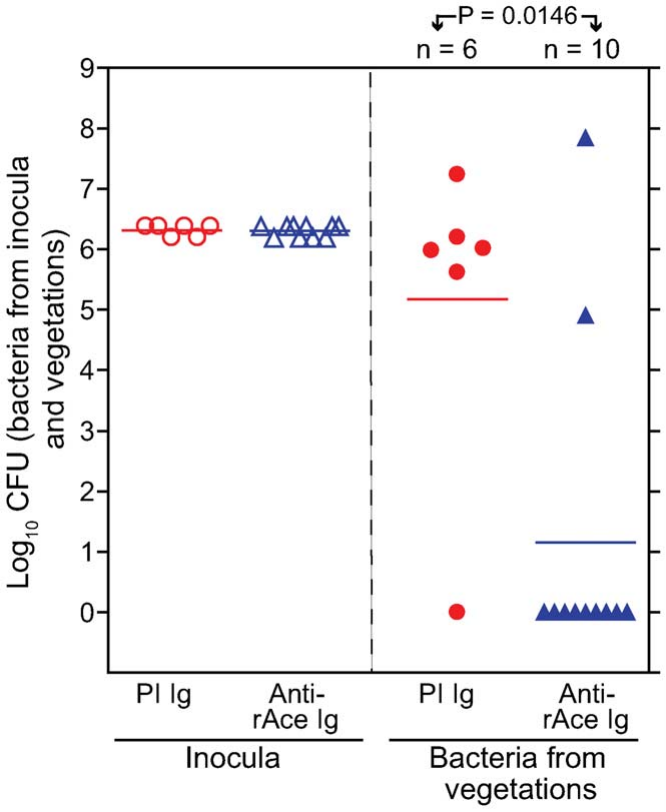
Passive immunization (anti-rAce Ig versus PI Ig) in rat endocarditis model. In the panel on the left, empty circles and empty triangles represent OG1RF inocula for pre-immune (PI) Ig treated and affinity purified specific anti-rAce Ig treated rats, respectively. In the panel on the right, solid circles and solid triangles represent OG1RF recovered from rat vegetations 24 h post infection. Horizontal bars represent the geometric means. Significantly fewer rats were infected by OG1RF in rAce Ig (2 mg/kg) (2/10) versus PI Ig (2 mg/kg) (5/6) treated rats (*P* = 0.0001 by Fisher's exact test). Rats (PI Ig treated) showed a mean ± SD increase of 3.8±1.4 log_10_ OG1RF CFU/gm from vegetations versus anti-rAce Ig treated rats (*P* = 0.0146 by unpaired *t* test).

Five of 6 (83%) of rats administered purified control Igs (2 mg/kg) from pre-immune serum 1 h prior to inoculation of OG1RF developed *E. faecalis* IE compared with 2/10 rats (20%) of the group given anti-rAce Igs (2 mg/kg) affinity-purified from immune serum (*P* = 0.0001) (Figure 8). Mean bacterial titers recovered from control rat aortic vegetations showed 3.8±1.4 log_10_ more CFU/gm than the group given anti-rAce Igs (*P* = 0.0146) (Figure 8). Thus, these results corroborated the protection results seen above in active immunization using rAce against *E. faecalis* IE in rats.

### Mouse peritonitis model

**Figure 9:**
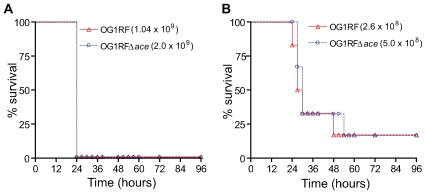
Kaplan-Meier survival plots of wild-type OG1RF and the *ace* mutant in the mouse peritonitis model. (A) Survival plots of OG1RF and OG1RFΔ*ace* using 10^9^ inocula (B) Survival plots of OG1RF and OG1RFΔ*ace* using 10^8^ inocula. Six mice were tested with each inoculum of each of the strains shown.

In this *in vivo* model, both OG1RF and OG1RFΔ*ace* caused animal mortality at similar rates with all the inocula tested (data with two inocula were shown in Figure 9) showing that OG1RFΔ*ace* was not attenuated versus OG1RF.

## Discussion

Infective endocarditis, which affects the endothelial lining of the heart, is among the most severe of the wide range of enterococcal infections encountered in humans, presenting a major therapeutic challenge and resulting in considerable mortality even when treated with antibiotics [5],[7],[8],[25]. Development of endocarditis can be initiated by injury to the valvular endothelium, which disrupts the normal valve structure and exposes underlying tissues, including ECM material. Deposition of host proteins, such as fibrin, as well as platelets at the site of injury then leads to the formation of a sterile thrombotic vegetation. This endovascular lesion may become colonized by circulating bacteria, leading to the growth of an infected vegetation. Valvular and aortic tissues are rich in collagen [26], and collagen is also found in sterile vegetations [26]. Previous studies have demonstrated that Ace plays a major role in the *in vitro* adherence of *E. faecalis* isolates to immobilized collagen [11],[12],[13]. Therefore, we reasoned that collagen could serve as a potential adhesion target for enterococci during bacteremia and that Ace could mediate bacterial attachment to these collagen-containing sites. To date, no studies have demonstrated a role for Ace in endocarditis and only very recently has a report appeared showing that Ace is important in a murine urinary tract infection model [27]. However, our previous demonstration of the role of host-derived cues (*i.e.*, using moieties typically encountered in the host, such as serum or collagen [19],[20]), for induction of both Ace expression and adherence of *E. faecalis* cells to collagen, suggested that both of these phenotypes are elicited by close association of this organism with a mammalian host/tissue. Moreover, we found that 90% of patients with prior *E. faecalis* endocarditis have Ace-specific antibodies in their sera, implying that Ace is expressed *in vivo* during the infection and that it is immunogenic [19]. For these reasons, we chose an experimental IE model in this study to explore the role of Ace in *E. faecalis* pathogenesis.

We first looked for direct evidence showing that Ace is expressed during *E. faecalis* infection. *E. faecalis* OG1RF cells recovered directly from infected vegetations showed surface expression of Ace with a much higher (∼3×) mean fluorescence intensity compared to cells grown *in vitro* in BHI at 37 °C, indicating that the host environment in vegetations, similar to collagen and serum [19],[20], can induce production of Ace and its localization on the cell surface.

As anticipated, *in vitro* ECM protein adherence results with OG1RFΔ*ace* corroborated our previous results with an insertionally inactivated *ace* [11],[20]. Collagen adherence of the *ace* deletion mutant was restored by complementation *in trans* and the adherence of the complemented strain was 1.5-fold above the level of the WT parent strain, likely due to the higher number of Ace molecules displayed on the surface of the complemented strain, as determined by flow cytometry.

Deletion of *ace* resulted in significant attenuation in the ability of the mutated *E. faecalis* OG1RF strain to compete successfully with its isogenic WT parent in infection of vegetations in a mixed-inoculum rat IE model. To the best of our knowledge, this is the first demonstration that Ace contributes to *E. faecalis* virulence in endocarditis. When we complemented the *ace* mutant *in trans*, significantly more colonization of heart valves was observed at 4 h after infection by this strain than by an isogenic strain containing an empty vector. Thus, these results confirmed our 72 h results with the *ace* deletion mutant and, furthermore, provide evidence that Ace plays an important role during the initial attachment and colonization stage of IE development, possibly by mediating adherence of *E. faecalis* cells to exposed collagen at the site of endovascular injury. This is consistent with the high surface expression levels of Ace in the complemented strain shown by our flow cytometry analysis. While stably maintained by the majority of *E. faecalis* cells during early colonization (4 h), the high instability of the complementation vector after extended growth in vegetations (94–98% of cells had lost the plasmid by 24 h) reduced its effect at later stages of endocarditis. The residual ability of OG1RFΔ*ace* to cause endocarditis in some rats indicates that Ace is not absolutely required for *E. faecalis* to cause endocarditis; this is in agreement with published studies that showed a role for additional factors in causing *E. faecalis* IE [28],[29],[30]. While the precise mechanism of action of Ace for initiating, maintaining and/or propagating IE has yet to be elucidated, we infer that the difference in virulence of OG1RFΔ*ace* may be due to its reduced ability to adhere to collagen. However, we cannot exclude the possibility of another ligand or another function of this protein.

Interestingly, deletion of *ace* did not result in observable effects in the mouse peritonitis model in terms of either time to death or total mortality, suggesting that *ace* is not important for this infection or that the direct administration of a large inoculum of bacteria into the peritoneal cavity may bypass an early infection stage where Ace might be involved. These results also indicate that deletion of *ace* did not affect growth or survival of OG1RF *in vivo* in general, consistent with the similar growth rate and viability of the *ace* deletion mutant and WT when grown *in vitro*.

We have recently shown that Acm, a collagen adhesin from *E. faecium*, is an important factor for endocarditis caused by that species [23]; this is similar to a previous observation with Cna of *S. aureus* [31], which is also involved in other infections, such as septic arthritis [32]. These MSCRAMMs share a large degree of sequence conservation in their collagen-binding domains; similar proteins are present in several other species of gram-positive pathogens, such as *Streptococcus equi* [33], *Arcanobacterium pyogenes* [34], *Bacillus anthracis* [35] and *Streptococcus gallolyticus* [36], and they possibly share a similar collagen-binding mechanism, called the “Collagen Hug” that has been characterized for Cna and Ace [15],[37]. Therefore, it seems plausible that this family of proteins has been preserved or acquired across different gram-positive species/genera as a generalized mechanism to provide a binding function, although the ligand in the ecological niches where enterococci are found in nature and the purpose for these adhesins is not known. Recently, we described Ebp pili as another important factor for *E. faecalis* endocarditis [29], as well as urinary tract infections and biofilm formation [29],[38], a further indication of the significance of surface proteins of the MSCRAMM family for *E. faecalis* pathogenesis.

Our results with active immunization of rats using the collagen-binding domain of Ace showed that only 25% of immunized rats developed endocarditis, while the infection rate in the untreated group was 100%. Protection was also evident when bacterial counts were evaluated. Consistent with these results, prophylactic treatment of rats with affinity-purified anti-Ace antibodies raised against the collagen-binding domain of Ace significantly reduced bacterial numbers in vegetations, demonstrating that passive transfer of Ace-specific antibodies confers significant protection against *E. faecalis* IE in rat. The differences in pre-infection procedures between the active- and passive-immunized groups preclude direct comparison of results from these two methods. Based on the results presented in this study, it seems likely that these preventive strategies specifically target the initial attachment and colonization stage of endocarditis by blocking collagen adherence of *E. faecalis* cells. Consistent with this hypothesis, we have previously shown that Ace-specific polyclonal antibodies purified from immunized rabbits or from humans with a prior *E. faecalis* endocarditis infection were effective in inhibiting adherence of Ace-expressing *E. faecalis* isolates to collagen [11],[19]. Furthermore, a recent study that generated monoclonal antibodies against rAce showed that some of the mAbs completely inhibited binding of rAce to collagen and Ace-coated fluorescent beads to epithelial cell lines [21].The *ace* gene is ubiquitously present among isolates of *E. faecalis* and its encoded amino acid sequence, especially within the collagen-binding domain, is highly conserved [19]. Therefore, targeting *ace* could potentially offer protective immunization against a large spectrum of genetically diverse *E. faecalis* isolates, an advantage over other virulence-associated factors, such as aggregation substance, hemolysin and gelatinase, which were found to be produced by <45% of endocarditis isolates [39] and for which protective efficacy has not been shown [40]. So far, only one *E. faecalis* antigen, the capsular polysaccharide, has shown promise as a potential vaccine candidate, as passive and active immunization against it lowered bacterial counts in kidneys, spleens and livers in a mouse *i.v.* infection model [41]. To our knowledge, our study is the first report of an immunization strategy that reduces *E. faecalis* colonization of aortic valves and shows protection against the development of *E. faecalis* endocarditis, thus, suggesting Ace as a promising alternative target for prophylaxis of *E. faecalis* endocarditis in high risk patients. However, the ability of OG1RF to cause IE in some of the rAce-immunized rats and also in some anti-Ace antibody-treated rats indicates that targeting multiple MSCRAMMs may be necessary for a robust protection of *E. faecalis* IE. Consistent with this, a recent study [42] showed full vaccine protection against abscess formation or lethal challenge with *S. aureus* strains when a combination of four MSCRAMM antigens were used versus a moderate reduction in bacterial load when used as individual vaccine antigens.

In summary, we have demonstrated here that i) deletion of the *ace* gene resulted in significant attenuation of the ability of *E. faecalis* to colonize aortic valves and cause endocarditis in an experimental rat IE model, coinciding (ii) with reduced *in vitro* adherence by the *ace* deletion mutant to collagen types I and IV; we have also shown that (iii) Ace is actively expressed within host vegetations during endocarditis and that (iv) both active and passive immunization against the collagen-binding domain of Ace conferred significant protection against endocarditis and reduced the numbers of bacteria found in vegetations. Taken together, these results demonstrate that Ace is an important virulence-associated factor and a likely target for prophylactic and therapeutic strategies against *E. faecalis* endocarditis. Since Ace-like proteins are widespread among streptococci and staphylococci, future cross-protection studies may reveal novel opportunities for the development of vaccines or immunotherapeutics that may be useful for the prevention and treatment of gram-positive infective endocarditis.

## Materials and Methods

### Ethics statement

The rat endocarditis model and surgical procedures were performed in accordance with the institutional policies and the guidelines stipulated by the animal welfare committee, University of Texas Health Science Center at Houston (AWC, UTHSC). This study was reviewed and approved by the University Institutional Review Board (AWC approval # HSC-AWC-08-067).

### Bacterial strains, plasmids, materials, standard molecular techniques, and growth conditions

*E. coli* and *E. faecalis* strains and all plasmids used in this study are listed in Table 1. All constructs were given TX numbers and plasmids from these constructs were assigned respective pTEX numbers (Table 1). *E. coli* strains were grown in Luria-Bertani media (Difco Laboratories, Detroit, Mich.). Enterococci were grown either in BHI, BHIS, Todd-Hewitt (TH) broth/agar (Difco Laboratories) or Enterococcosel™ Agar (EA) (Becton Dickinson) at 37°C, unless a different growth temperature is specified. The following antibiotic concentrations were used with *E. faecalis*: chloramphenicol 10 µg/ml, kanamycin 2000 µg/ml, rifampicin 100 µg/ml and gentamicin 125 µg/ml. With *E. coli*, the concentrations used were chloramphenicol 10 µg/ml, kanamycin 50 µg/ml, and gentamicin 25 µg/ml. Resistance of enterococci to gentamicin and kanamycin was defined as MICs >500 and >2000 µg/ml, respectively [18].

### Materials

All antibiotics were obtained from Sigma (St. Louis, Mo.). Tran ^35^S-label and bovine serum albumin (BSA) were purchased from MP Biomedicals Inc. (Irvine, Calif.). C I and CIV were from Sigma and Fg was from Enzyme Research Laboratories (South Bend, Ind.). Oligonucleotide primers were purchased from Invitrogen (Carlsbad, Calif.) or IDT (Coralville, Iowa) or Sigma and their sequences are provided in Table 2. Restriction enzymes and DNA modification enzymes were mostly from Invitrogen and New England BioLabs, Inc. (Beverly, Mass.). All other chemicals used in the investigation were of molecular biology grade.

**Table 2:** Primers used in this study.

Primer Name	Sequence (5′→3′)^a^	Function	Amplicon
AceDelF1	CGCGGATCCTGCCGAGTGACAGGCATTCTGTATTGC	Deletion mutant generation	Upstream fragment of *ace*
AceDelR1	CCCAAGCTTCTCTTATTTTTTCCACTTAGTGGTCTT	Deletion mutant generation	Upstream fragment of *ace*
AceDelF2	AAAACTGCAGTTTCTATTATCTGGAGATAAATTGCTG	Deletion mutant generation	Downstream fragment of *ace*
AceDelR2	CCGGAATTCTTCCAAGCGCTGATAGGCTACTTTATC	Deletion mutant generation	Downstream fragment of *ace*
AceUpF1	CCAAACATATTGCCACTTAAATCTCTA	Mutant confirmation	
AceDnR1	CACACATCTTTTAATGAAATTGTTTGA	Mutant confirmation	
AceComF1	GCGGAGCTCAGAAGGGTGAATAATTTTTTATGAC	Complementation	Complete *ace*
AceComR1	GCGGGATCCTTAATTCTTTCTGATTTGTAGATAAC	Complementation	Complete *ace*

^*a*^Introduced restriction sites are underlined.

### Standard molecular techniques

Chromosomal DNA from *E. faecalis* isolates was prepared following the hexadecyltrimethyl ammonium bromide method described earlier [43]. Plasmid DNA was isolated from *E. coli* using the Wizard Plus SV minipreps DNA purification system (Promega Corporation, Madison, Wis.) and, from *E. faecalis*, by a previously described method [44]. General recombinant DNA techniques such as ligation and agarose gel electrophoresis were performed using standard methods [45]. When necessary, DNA fragments were purified with low melting temperature agarose gel followed by purification using QIAquick-gel extraction kit (Qiagen Inc., Valencia, Calif.). PCR reactions were performed with a Perkin-Elmer GeneAmp PCR system 9700 using the optimized buffer B (1 × buffer: 60 mM Tris-HCl [pH 8.5], 15 mM ammonium sulfate and 2 mM MgCl_2_) obtained from Invitrogen. PCR-generated fragments were purified using the Wizard PCR DNA Cleanup System (Promega Corporation). Recombinant plasmids were generated in *E. coli* DH5α or XL1-blue. Electroporation of *E. coli* and *E. faecalis* was carried out using a Gene Pulser (Bio-RAD Laboratories, Richmond, Calif.) as described previously [46],[47]. Agarose plugs containing genomic DNA were digested with SmaI and PFGE was performed using a previously described method [1]. Southern blotting was performed using Hybond-N^+^ nylon membrane and 0.4 N sodium hydroxide solution. Preparation of colony lysate blots was described elsewhere [48]. The RadPrime DNA Labeling System (Invitrogen) was used for labeling DNA probes with [α-^32^P] dCTP (GE Healthcare, Piscataway, N.J.) and hybridizations were carried out using high stringency conditions [48],[49]. DNA sequencing reactions were performed by the *Taq* dye-deoxy terminator method and an automated ABI Prism sequencer (Applied Biosystems, Foster city, Calif.).

### Construction of an *ace* deletion mutant in OG1RF and its complementation

An *E. faecalis ace* mutant (OG1RFΔ*ace*::*cat*) was constructed by allelic replacement using pTEX5500ts as described earlier for *E. faecium* [50]. We used a replacement strategy in this study to facilitate distinguishing between WT and OG1RFΔ*ace* during *in vivo* animal experiments with mixed cultures; bioinformatics and mRNA analyses of the *ace* locus predicts the absence of a polar effect of *ace* deletion by the *cat* replacement (unpublished data). *E. faecalis* OG1RFΔ*ace* was constructed by allelic replacement using pTEX5500ts as described earlier for *E. faecium* [50]. A 1027-bp DNA fragment (AceDelUp) encompassing the region upstream of *ace* was amplified from OG1RF genomic DNA template using primers AceDelF1 and AceDelR1 (Table 2), digested with BamHI and HindIII, and ligated with similarly digested pTEX5500ts. Similarly, a 989-bp DNA fragment (AcedelDn) encompassing the region downstream of *ace* was amplified from the same genomic DNA template using primers AceDelF2 and AceDelR2 (Table 2). The AceDelDn PCR product digested with PstI and EcoRI was ligated to similarly digested pTEX5500ts::AceDelUp and was then transformed into *E. coli* DH5α to obtain TX5428. The plasmid from this construct, pTEX5428 (pTEX5500ts::AceDelUp+AceDelDn), was introduced into electrocompetent cells of OG1RF and cells were then plated on gentamicin plates at the permissive temperature (28°C). A single gentamicin and chloramphenicol resistant colony from these plates was grown overnight at 42°C, then plated on chloramphenicol plates and incubated at 37°C. After confirming the specific single crossover integration (OG1RF*ace*Up::pTEX5428) by PCR (with primer sets AceUpF1 and CmR as well as AceDnR1 and CmF), one of the integrants was picked, grown for eight overnight serial passages at 42°C, and then plated on BHI to select for plasmid excision by double crossover recombination. The colonies from these BHI plates were then replica plated to chloramphenicol plates and gentamicin plates to identify colonies that retained the *cat* gene but not the vector.

To complement OG1RFΔ*ace in trans*, an ∼2 kb fragment containing the *ace* open reading frame plus its ribosome-binding site (amplified using primers *ace*ComF1 and *ace*ComR1; Table 2) was cloned under the control of the P2 promoter of the shuttle vector, pAT392 [51]. This *in vitro*-ligated construct for complementation (designated as pTEX5646) was transformed into *E. coli* XL1-Blue to obtain TX5646 and was then introduced into electrocompetent cells of TX5467 to obtain TX5647 (OG1RFΔ*ace* (pAT392::*ace*). Surface expression of Ace in OG1RFΔ*ace* (pAT392::*ace*) was determined by flow cytometry (see below).

### Growth curves

Overnight cultures were inoculated into BHI broth at a dilution of 1:100. The cultures were then grown at 37°C with shaking in an orbital shaker and aliquots were removed hourly from 0 to 12 h and at 24 h, for determining the absorbance at 600 nm (OD_600_) with a spectrophotometer.

### *In vitro* adherence assay

Adherence of *E. faecalis* to CI, CIV, Fg and BSA was determined in four independent experiments using Tran ^35^S-labeled bacteria by a previously described assay [11].

### Expression and purification of (His)_6_ tagged Ace A domain

Construction of the recombinant plasmid pTEX5254 (complete *ace* A domain of OG1RF cloned into pBAD/HisA expression vector) was described previously [11]. Expression cultures of TX5254 were induced with arabinose and the N-terminally His_6_ tagged proteins were purified using nickel affinity chromatography and anion exchange chromatography, as described previously [11],[52]. Protein concentrations were determined by absorption spectroscopy at 280 nm using calculated molar absorption coefficient values [53].

### Ace specific polyclonal antibodies

Expression and purification of (His)_6_-tagged recombinant Ace A domain was done using a previously described construct and methods [11]. Goat polyclonal serum against recombinant rAce A domain (rAce) was generated by Bethyl Laboratories (Montgomery, TX). Ace A-domain specific antibodies were eluted from rAceA coupled to cyanogen bromide-activated Sepharose 4B, according to the manufacturer's protocol (Amersham Biosciences, Piscataway, N.J.). The antibodies were concentrated by ultrafiltration with a 10,000-Da molecular-weight-cutoff filter (Millipore, Bedford, Mass.), dialyzed against PBS and concentrations were determined by absorption spectroscopy.

### Protein extraction and Western blotting

Surface protein extracts from *E. faecalis* isolates were prepared using mutanolysin (Sigma) as described earlier [11]. Protein extracts were electrophoresed in 4–12% NuPAGE Bis-Tris gels (Invitrogen) under reducing conditions in MOPS buffer, and transferred to a polyvinylidene difluoride (PVDF) membrane. Membranes were then incubated with either affinity-purified anti-Ace A-domain specific immunoglobulins (Igs) [11] or pre-immune rabbit serum Igs followed by horseradish peroxidase-conjugated anti-goat antibodies. The blots were then developed with Supersignal West Pico Chemiluminescent substrate (PIERCE, Rockford, Ill.). Purified recombinant Ace A-domain was used as a positive control.

### Flow cytometry

#### Bacteria

Surface expression of Ace on OG1RF or OG1RFΔ*ace* (pAT392::*ace*) cells was detected by flow cytometry using affinity purified Ace A-domain (ligand-binding domain)-specific antibodies, as described earlier [54]. Bacteria grown for 10 h in appropriate conditions were probed with pre-immune or affinity purified anti-rAceA specific-antibodies followed by donkey anti-goat IgG F(ab')_2_ fragment conjugated with *R*-phycoerythrin (Jackson Immunoresearch Laboratory, West Grove, Pa.). The cells were fixed in paraformaldehyde and analyzed with a Coulter EPICS XL AB6064 flow cytometer (Beckman Coulter, Fullerton, Calif.) and System II software.

#### Endocarditis vegetations

Sterile vegetations were produced in 11 rats, as described below, and nine rats were injected (*i.v.*) with OG1RF. Vegetations harvested after 48 h from the nine infected and two non-infected rat heart valves were processed to remove host tissue debris, using a previously described method [23]. Processed samples in groups of three infected vegetations were mixed and then divided into aliquots for labeling with a) Igs purified from antiserum raised against formalin-killed *E. faecalis* HH22-whole-cells (positive control) and b) affinity-purified anti-rAce-specific Igs. To assess possible cross-reactivity of anti-rAce Igs with host tissue, the non-infected vegetation sample was probed with affinity-purified anti-rAce-specific Igs. Forward scatter (for analysis of particle sizes in the sample) and side scatter (for analysis of cell granularity or internal complexity) of vegetation processed cells were analyzed and compared with the *in vitro* grown *E. faecalis* OG1RF cells.

### Antibody titers

Anti-Ace antibody titers in rat sera were determined by ELISA as described by [19] with some modifications. Briefly, 96-well plates (Immulon 4HBX, Thermo Fisher Scientific, Waltham, Mass.) were coated with 1 μg of rAce in 0.05 M carbonate-bicarbonate buffer, pH 9.6. Rat sera were tested in duplicate with serial dilutions from 1∶100 to 1∶240,800, followed by detection with peroxidase-conjugated anti-rat secondary antibody (Jackson ImmunoResearch Laboratory, West Grove, Pa.) and TMB peroxidase substrate (Bethyl Laboratories, Montgomery, Tex.). The reaction was stopped with 2 M H_2_SO_4_. Antibody titers were expressed as the highest serum dilution with an A_450nm_ ≥0.10 at 3 min after addition of the substrate [55].

### Testing the effect of *E. faecalis* OG1RF, *ace* mutants, complemented mutants and immunizations in experimental endocarditis

Aortic valve endocarditis was produced in rats by following previously published methods [23],[30],[55],[56],[57],[58]. In brief, for induction of endocarditis, white Sprague-Dawley rats (∼200 gm) were used. The animals were anesthetized with isoflurane for placement of intravascular catheters. The right carotid artery was exposed and a sterile polyethylene catheter was inserted through a small incision and advanced to ∼4 cm into the left ventricle. Proper positioning was assured by sensing resistance and vigorous pulsation of the line.

#### Mixed infection competition assay using OG1RF and *ace* mutants

For testing OG1RF and OG1RFΔ*ace* in a mixed infection competition assay, bacteria (∼1∶1 by OD) were inoculated via the catheter 20 min after catheter placement; the catheter was heat-sealed and left in place during the course of the experiment and the skin was closed with sutures. Bacterial geometric mean (GM) CFUs determined for OG1RF and OG1RFΔ*ace* from the inocula were 3.8×10^7^/rat and 4.4×10^7^/rat, respectively. Animals were sacrificed 72 h after infection. Hearts were aseptically removed and aortic valves were examined. The platelet-fibrin vegetations formed on aortic valves were excised, weighed, homogenized in 1 ml of saline and dilutions were plated onto BHI and EA media. Ninety-six colonies/rat were picked into microtiter plate wells containing BHI, grown for 4–6 h and then replica plated onto EA and BHI supplemented with chloramphenicol 10 µg/ml, or BHI supplemented with kanamycin 2000 µg/ml in the case of the *ace* disruption mutant, to verify the phenotypic markers of OG1RF and OG1RFΔ*ace*. DNA lysates from colonies obtained from the OG1RFΔ*ace* plus OG1RF mixed infection were hybridized under high stringency conditions [48], using intragenic DNA probes of *ace* and *cat* in order to generate the percentage (%) of OG1RF and OG1RFΔ*ace* colonies of the recovered bacteria from vegetations. Rats with sterile cultures of undiluted vegetation homogenates (∼1 ml) were considered to have had no induction of endocarditis. Data were expressed as percentages (%) of WT and mutant per vegetation. For vegetations showing only OG1RF colonies, for example, when 96/96 colonies were *cat* negative and *ace* positive and 0/96 colonies were *cat* positive and *ace* negative, the proportion of OG1RFΔ*ace* in the vegetation was assigned the value of 1/97 (∼1%) (*i.e*., assuming the next colony picked would have been a mutant).

#### Mono-infection testing using complemented OG1RFΔ*ace* (pAT392::*ace*) and OG1RFΔ*ace* (pAT392)

For testing of OG1RFΔ*ace* (pAT392::*ace*) versus OG1RFΔ*ace* (pAT392) in the mono-infection model, rats were inoculated (*i.v.*) 24 h post-catheterization and were sacrificed at 4 h or 24 h post infection. Due to the instability of pAT392 seen in *in vivo* experiments, inocula were then grown in the presence of gentamicin (125 μg/ml) and animals were sacrificed at 24 h post infection. To minimize the loss of pAT392 over time and to determine the role of Ace in early colonization of aortic valves by OG1RFΔ*ace* (pAT392::*ace*) versus OG1RFΔ*ace* (pAT392), we also sacrificed a group of 11 to 12 animals administered with bacteria grown in BHIS at 4 h post inoculation. Rats with sterile cultures of undiluted vegetation homogenates were considered to have had no induction of endocarditis. In order to determine the *in vivo* stability of plasmid pAT392 in complemented OG1RFΔ*ace* (pAT392::*ace*) and OG1RFΔ*ace* (pAT392), ∼95 random colonies recovered from vegetations were picked into microtiter wells and were replica plated onto BHI supplemented with gentamicin 125 μg/ml versus BHI to differentiate between gentamicin resistant (the marker of pAT392) [51] and gentamicin susceptible colonies.

#### Estimation of WT OG1RF ID_50_ in rat IE model

ID_50_ of OG1RF was determined for BHI and BHIS grown cultures. Twenty one rats were inoculated (*i.v.*) with either BHI or BHIS grown OG1RF in a range of 1.6×10^4^–2.6×10^8^ and 7.2×10^5^–1.1×10^9^ CFU/ rat, respectively. The ID_50_ was determined by the method of Reed and Muench [59].

#### Immunizations

For active immunization, animals were divided into three groups: (a) rAce active-immunized, (b) non-immunized and (c) FCA – FICA sham-immunized. Immunizations were done following previously published methods [55],[56]. In brief, animals in group (a) received an initial dose of 100 μg of rAce in FCA (week 1) followed by a second and third dose of 100 μg of rAce in FICA at a two weeks interval. Animals in group (c) received FCA (week 1) followed by a second and third dose of FICA at a two weeks interval. Surgeries and catheter placement were done as described above for testing the *ace* mutant. Twenty-four hours post catheterization, animals were inoculated (*i.v.*) using BHI- or BHIS-grown OG1RF and were sacrificed at 48 h post infection. Three independent experiments were done and results were combined.

For the passive immunization group, animals were injected (*i.v.*) with 2 mg/kg of affinity purified Ace Igs purified from immunized goat serum 24 h post-catheterization and 1 h prior to bacterial inoculation; controls received 2 mg/kg of purified Igs from pre-immune goat serum. Animals were sacrificed at 24 h post infection. Two independent experiments were done and results were combined.

#### Post euthanasia procedures

Hearts were aseptically removed from all euthanized animals. The vegetations on aortic valves were excised, weighed, homogenized in 1 ml of saline and dilutions were plated onto BHI, EA and BHI supplemented with rifampicin 100 µg/ml for bacterial CFU recovery. Rats with sterile cultures of undiluted vegetation homogenates were considered to have had no induction of endocarditis.

### PFGE

Randomly picked colonies recovered from vegetations of infected rats were also tested by PFGE to reconfirm the infecting organism [49]. In brief, agarose plugs containing genomic DNA were digested with SmaI and PFGE was performed using a previously described method with ramped pulse times of 5 s and 45 s.

### Mouse peritonitis model

OG1RF and OG1RFΔ*ace* were tested following our previously published method [60]. In brief, mice were injected intraperitoneally with appropriate dilutions of bacteria (BHI or BHIS), premixed with sterile rat fecal extract (SRFE) and were observed for 5 days for survival. Two-fold inocula (range of ∼1×10^8^–1×10^9^ CFU/ml) of both test bacteria were used to compare animal survival/mortality. LD_50_ was determined using six mice per group and by the method of Reed and Muench [59].

### Statistics

To compare the mean ± SD values of the adherence results, an unpaired *t* test was used. Percentages (%) of OG1RF versus OG1RFΔ*ace* present in mixed infection vegetations were analyzed by the paired *t* test. Similar to the method previously described for *E. faecalis* and *E. faecium* endocarditis using a mixed infection [23],[29], the mean virulence index of the mutant relative to WT was calculated using the following equation:
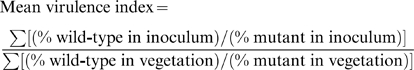



Mean virulence index for the mutant should be 1.0, if the WT and the mutant have the same level of virulence, and lower values would indicate increasing levels of attenuation. Differences in bacterial log_10_ CFU (geometric mean) from vegetations of rAce-immunized versus non-immunized and FCA-FICA-immunized controls were analyzed by the unpaired *t* test. Fisher's exact test was used for comparing the total number of infected/non-infected rats in the rAce-immunized group versus control groups. Graph Pad Prism version 4.00 for Windows (GraphPad Software, San Diego, Calif.) was used for statistical analysis.
