# Correction: Importance of the Collagen Adhesin Ace in Pathogenesis and Protection against *Enterococcus faecalis* Experimental Endocarditis

**DOI:** 10.1371/annotation/1ccae8f8-d274-4ff8-a295-815037ce9cc6

**Published:** 2010-02-01

**Authors:** Kavindra V. Singh, Sreedhar R. Nallapareddy, Jouko Sillanpää, Barbara E. Murray

A competing interest statement was erroneously omitted for the 2nd and 4th authors, Sreedhar R. Nallapareddy and Barbara E. Murray. The Competing Interests section should read: Drs. Barbara E. Murray and Sreedhar R. Nallapareddy are co-inventors of useful improvements in COLLAGEN-BINDING PROTEINS FROM ENTEROCOCCAL BACTERIA, a patent for which the rights have been assigned to The Texas A&M University System (College Station, TX) and the Board of Regents of the University of Texas System (Austin, TX, USA).

